# *In Vitro*–*In Vivo* Extrapolation by Physiologically Based Kinetic Modeling: Experience With Three Case Studies and Lessons Learned

**DOI:** 10.3389/ftox.2022.885843

**Published:** 2022-07-18

**Authors:** Engi Abdelhady Algharably, Emma Di Consiglio, Emanuela Testai, Francesca Pistollato, Hans Mielke, Ursula Gundert-Remy

**Affiliations:** ^1^ Charité—Universitätsmedizin Berlin, Corporate Member of Freie Universität Berlin and Humboldt-Universität zu Berlin, Institute of Clinical Pharmacology and Toxicology, Berlin, Germany; ^2^ Mechanisms, Biomarkers and Models Unit, Environment and Health Department, Istituto Superiore di Sanità, Rome, Italy; ^3^ European Commission, Joint Research Center (JRC), Ispra, Italy; ^4^ Federal Institute for Risk Assessment, Berlin, Germany

**Keywords:** reverse dosimetry, risk assessment, toxicodynamics, toxicokinetics, new approach methodologies (NAMs)

## Abstract

Physiologically based kinetic (PBK) modeling has been increasingly used since the beginning of the 21st century to support dose selection to be used in preclinical and clinical safety studies in the pharmaceutical sector. For chemical safety assessment, the use of PBK has also found interest, however, to a smaller extent, although an internationally agreed document was published already in 2010 (IPCS/WHO), but at that time, PBK modeling was based mostly on *in vivo* data as the example in the IPCS/WHO document indicates. Recently, the OECD has published a guidance document which set standards on how to characterize, validate, and report PBK models for regulatory purposes. In the past few years, we gained experience on using *in vitro* data for performing quantitative *in vitro*–*in vivo* extrapolation (QIVIVE), in which biokinetic data play a crucial role to obtain a realistic estimation of human exposure. In addition, pharmaco-/toxicodynamic aspects have been introduced into the approach. Here, three examples with different drugs/chemicals are described, in which different approaches have been applied. The lessons we learned from the exercise are as follows: 1) *in vitro* conditions should be considered and compared to the *in vivo* situation, particularly for protein binding; 2) *in vitro* inhibition of metabolizing enzymes by the formed metabolites should be taken into consideration; and 3) it is important to extrapolate from the *in vitro* measured intracellular concentration and not from the nominal concentration to the tissue/organ concentration to come up with an appropriate QIVIVE for the relevant adverse effects.

## 1 Introduction

Physiologically based kinetic (PBK) modeling has been increasingly used since the beginning of the 21st century to support dose selection of drugs to be safely applied in the first studies in humans (Poulin and Theil, 2002). For chemical safety assessment, the potential use of PBK models is still less frequently applied for refining animal-to-human or route-to-route extrapolations. Back in 2010, in an IPCS/WHO document, most of the PBK models were based mainly on *in vivo* data. Recently, the OECD has published a guidance document which sets standards for the characterization, validation, and reporting of PBK models for regulatory purposes, which also emphasizes the use of *in vitro* and *in silico* approaches in PBK model development (OECD, 2021). Currently, in the context of next-generation risk assessment of chemicals, the application of new approach methodologies (NAMs) together with PBK has been playing an important role (Coecke et al., 2013; EFSA, 2014; SCCS, 2021). In order to improve the regulatory acceptance of NAM data, the human relevance of the alternative test together with the use of integrated approaches has become crucial for concluding the safety assessment of chemicals. Indeed, in recent years, the interest has focused on the potential of human cell-based *in vitro* testing to provide an animal-free approach assessing the risk arising from the exposure toward chemicals (Loizou, 2016; Punt et al., 2018; Paini et al., 2019). Approaches able to extrapolate *in vivo* doses from *in vitro* measured/estimated concentrations are fundamental in order to exactly define relevant *in vivo* exposures related to the observed *in vitro* adverse effects. The challenge of this approach, named quantitative *in vitro*-to-*in vivo* extrapolation (QIVIVE), is extrapolating from *in vitro* concentration-response data to *in vivo* safe exposures and/or to identify the exposure levels causing adverse effects (EFSA, 2014). Overall, such approaches will accelerate the future implementation of NAMs at the regulatory level, following the 3R principle (replacement, reduction, and refinement). At present, the *in vitro* non-observed and the observed adverse effects are generally correlated with the nominal concentration of the test item or at best with the concentration in the culture medium. This is a shortcoming, and several examples exist in the literature showing the difference between nominal and actual concentrations (Groothuis et al., 2015; Kramer et al., 2015; Pomponio et al., 2015a; Truisi et al., 2015). Furthermore, in many examples for the application of QIVIVE, the underlying assumption is that the relationship between *in vitro* extra- and intracellular concentration is the same as the *in vivo* relationship between blood/plasma and intracellular concentrations in the relevant tissue; however, this might be erroneous (Mielke et al., 2017; Algharably et al., 2019).

Fit-for-purpose QIVIVE analyses require consideration of several key factors, including *in vitro* biokinetics, PBK modeling, *in vivo* (human) exposure parameterization, and the selection of adequate *in vitro* toxicodynamic assays to characterize the toxicity profile of a chemical (Parish et al., 2020). The selection of an adequate *in vitro* toxicodynamic assay is really challenging in cases where the toxicological profile is unknown. High-throughput data have been produced with many measured endpoints; however, it is not an easy task to select the relevant endpoints from a whole array of *in vitro* tests, which indicate toxicity of the substance in the *in vivo* situation without knowledge on the toxicological profile (Honda et al., 2019).

The aim of this study was to present results and share the experience gained in evaluating to which extent drug-induced side effects or chemical-induced adverse effects could be quantitatively predicted starting with *in vitro* data. For this purpose, we selected examples from the literature in which *in vitro* kinetics have been measured along with toxicodynamic effects. Nevertheless, knowledge about the mode of action (MoA) or the adverse outcome pathway (AOP) of the measured *in vitro* effects was not a selection criterion, being out of scope here. Indeed, for most of the selected effects, an AOP is not presently available in the OECD web page (https://aopwiki.org/oecd_page), and a comprehensively established MoA is not defined. QIVIVE predictions were subsequently compared with observed adverse effects when the drugs were clinically used or with the reported toxic effects by epidemiological data, following exposure to the chemical. In the current article, we presented three examples (ibuprofen, amiodarone, and chlorpyrifos) with different kinetic and dynamic behaviors. *In vivo* human concentration–time profiles of ibuprofen and amiodarone available in the literature allowed for a validation of the predicted concentration–time profiles. Chlorpyrifos concentration‐time profiles in non-pregnant women were available, however not in pregnant women; the target population for the toxicodynamic effect under investigation. For all the examples, we could compare the extrapolated doses from in vitro effect assays with doses which in humans were associated with adverse health effects.

## 2 Materials and Methods

### 2.1 Case 1 [Ibuprofen, IBU]

#### 2.1.1 Scenario

Data from an *in vitro* biokinetic study (Truisi et al., 2015) were the basis to perform a human QIVIVE (Mielke et al., 2017). In this *in vitro* biokinetic study, the time course of IBU concentration was measured in the cell culture medium and in the cell lysate of *in vitro* long-term cultures of primary human hepatocytes (PHH) after 14 days of repeated administration of IBU. The measured concentration in the medium and in the cells was considered to represent the plasma/blood concentration and the bioavailable concentration at the target organ for toxicity, that is, the liver, respectively. The selected *in vitro* testing dose level corresponded to the TC10, that is, the concentration at which 10% cytotoxicity was observed and hence equivalent to an *in vivo* human dose at which low degree toxicity could occur.

#### 2.1.2 Model Structure and Physiological Parameter Values

The human PBK (h-PBK) model was based on a structural model with eight organs/tissues and arterial and venous blood, the most important organ being the liver as the target for IBU toxicity. The organs were connected to the systemic circulation *via* arterial inflow and venous outflow; the circulation system was closed *via* the lung and the heart. The physiological data (organ weights, cardiac output, and organ blood flow) and characteristics of an adult male were taken from Abraham et al. (2005) .

Ibuprofen is a nonsteroidal anti-inflammatory drug with a low-molecular weight (206.27 g/mol), and tissue membranes do not represent a significant barrier to model the *in vivo* doses, leading to the *in vitro* measured concentrations.

#### 2.1.3 Drug-Specific Parameters

Physicochemical properties of IBU were retrieved from PubChem, log *P*_O/W_ = 2.23 (Hansch et al., 1995) and a non–protein-bound fraction of 0.01 (Cristofoletti and Dressman, 2014). Oral absorption was assumed to be 100% (Cristofoletti and Dressman, 2014). The tissue/blood partition coefficients were calculated according to Schmitt (2008a) and Schmitt (2008b). A further calculation was performed for the tissue/blood partition coefficient by using the experimental *in vitro* data (concentration in the cell lysate/concentration in the medium). Metabolism was assumed to be complete in the liver, and drug clearance was assumed to be by hepatic metabolic clearance only since the parent compound is not excreted by the kidney and was taken from 1) an *in vivo* study in human male volunteers (Greenblatt et al., 1984) and 2) the *in vitro* data in PHH, as reported in Truisi et al. (2015). Relevant model input parameters are summarized in Table 1.

**TABLE 1:** Selected physicochemical and physiological human PBK model input parameters for the three tested compounds.

	IBU	AMI	CPF
**Relevant physicochemical parameter**			
Molecular weight (g/mol)	206.29	645.31	350.57
Log*P*o: w	2.23	7.57	4.96
p*K*_a_	4.5	9.08	Non-dissociable
Solubility (mg/ml)	0.0684	0.00476	0.0014
**Relevant pharmacokinetic input parameter**			
Absorption	*k*_a_ 2.1 h^−1^ ^a^; *f*_a_ 100^a^	*k*_a_ 0.323 h^−1^ ^f^; *f*_a_ 0.3^f^	P_eff_ 6.16 e^−5^ cm/s^l^; *f*_a_ 0.32 (Calculated *in silico*)
Distribution			
fu	0.01	0.01–0.06	0.03
Tissue/blood partition coefficient	Calculated *in silico* ^b^ and for liver/blood also from *in vitro* data^c^	Calculated from rat *in vivo* data^g^	Calculated *in silico* ^b^
Clearance (hepatic)			
*In vivo* (L/h)	4.38^d^		Calculated *in silico* based on *in vitro V*_max_ and *K*_m_ ^m^
*In vitro*	*V*_max_/*K*_m_ 6.5 (μm^3^/s)^c^	*V*_max_ 4.12 (nmol/h/mg)^h^ *K*_m_ 38.85 (μM)^h^	*V*_max,_ _*Dearylation*_ 14.04 (nmol/min/mg)
			*V*_max,_ _*Desulfuration*_ 9.36 (nmol/min/mg)
			*K*_m,__*Dearylation*_ 4.33 (μM)
			*K*_m,__*Desulfuration*_ 28.5 (μM)
Scaled clearance	3.6 (L/h/kg bw)^e^	CL _*to MDEA*_ (main metabolite) 5.068 L/h^i^	Intrinsic clearance scaled *in silico* ^n^
		CL _*to*_ _*other metabolites*_ 0.096 L/h^j^
		CL _*to MDEA*_ 0.002 L/h^k^

P_eff_, specific intestinal permeability; CL, clearance; *k*_*a*_, first-order absorption rate constant; *f*_*a*_, fraction absorbed; fu, fraction unbound; MDEA, monodesethylamiodarone.

a Taken from Cristofoletti and Dressman, (2014).

b According to Schmitt, (2008a; Schmitt, 2008b).

c Truisi et al.(2015).

d Taken from Greenblatt et al. (1984).

e Scaled using Barter et al. (2007).

f Taken from Kannan et al. (1982).

g Taken from Lu et al. (2016).

h Taken from Trivier et al. (1993).

i Scaled with data taken from Trivier et al. (1993) using Barter et al. (2007).

j Taken from Chen et al. (2015).

k Scaled with data taken from Pomponio et al. (2015a) using Barter et al. (2007).

l Taken from Cook and Shenoy, (2003).

m Taken from Zhao et al. (2019).

n According to Rostami-Hodjegan and Tucker, (2007).

#### 2.1.4 Program

For the simulation of the whole body, all mass balance equations were combined in a system of interdependent differential equations. The rate of the change in concentration in every tissue was described by a system of time-dependent differential equations, with excretion by metabolism in the liver *via* constant clearance. RBC/cell partition and microsomal protein binding were not taken into account.

Distribution of the drug was modeled assuming drug uptake into the organs to be limited by the organ blood flow rate and organ capacity (i.e., organ volume and partition coefficient) and not linked to permeability, assuming well-stirred organs/tissues.

The program code was written in MATLAB (code available on request from the authors). Simulation was performed using MATLAB (version R2015b). The aim of the modeling was finding the *in vivo* dose in which *in vivo* dose corresponds to the *in vitro* TC10 in the PHH cell culture.

As *in vivo* data were available, no formal validation was performed. Instead, the predictivity of the h-PBK model was evaluated by visually comparing the predicted concentration–time profile in blood with plasma concentration–time data, as reported by Greenblatt et al. (1984) in an elderly volunteer after oral intake of a single dose of 600 mg IBU. In this evaluation process, we used the results obtained by using the hepatic partition coefficient calculated by the algorithm of Schmitt (2008a) and Schmitt (2008b) and, alternatively, the coefficient determined by using *in vitro* data (Truisi et al., 2015). In the same way, we used the clearance taken from *in vivo* data or calculated from the *in vitro* data (Truisi et al., 2015).

#### 2.1.5 Dose Finding by QIVIVE

To determine the *in vivo* dose which would generate the same concentration–time profile as *in vitro* (concentrations in the medium vs. the one in the cell lysate) after single exposure, we simulated the concentration–time profile in blood vs. the liver in an iterative process by varying doses in steps of 10 mg given to the human. The decision for the optimized dose was made by comparing the sum of squared (SSQ) differences, simulated vs. observed and decided to take the dose, which resulted in the lowest SSQ. In a second series, we determined the dose which best fitted the concentrations and measured in the cells because this was the relevant metric related to hepatic toxicity.

### 2.2 Case 2 [Amiodarone, AMI]

#### 2.2.1 Scenario

We used the available *in vitro* biokinetic data on AMI (Pomponio et al., 2015a; Pomponio et al., 2015b) and applied the QIVIVE approach in two scenarios related to 1) hepatotoxicity (Algharably et al., 2019) and 2) neurotoxicity (Algharably et al., 2021) as the two main possible adverse reactions elicited by AMI.

Scenario 1: using the data from an *in vitro* biokinetic study in PHH (Pomponio et al., 2015a), QIVIVE was performed to predict the *in vivo* AMI dose in man, leading to the same concentration–time profile obtained in the *in vitro* study in both the medium and hepatocytes after single and repeated exposure to a nominal concentration of 2.5 μM (concentration at which 10% cytotoxicity was observed). The aim of this simulation was to evaluate the relevance of the selected *in vitro* system and its power to predict the *in vivo* kinetics.

Scenario 2: the same approach was used to predict the *in vivo* doses that would result in the intracellular brain concentrations corresponding to that obtained in an *in vitro* biokinetic study (Pomponio et al., 2015b) after repeated AMI administration in rat brain cell culture. In this study, 3D re-aggregating brain cells were repeatedly treated for 14 days with AMI at two nominal concentration levels, 1.25 and 2.5 μM, that were associated with statistically significant changes in some markers of neurotoxicity (e.g., neuronal insult, cellular stress, cytotoxicity, and functional markers). For the dynamic effects, a dose-response was studied with four nominal concentrations 0.312, 0.625, 1.25, and 2.5 μM. In analogy to the first study, actual concentrations of AMI and its main metabolite monodesethylamiodarone (MDEA) were measured both in the medium and the cells. For QIVIVE, we primarily used the area under the concentration–time curve (AUC) over 24 h as the target metric for the reverse dosimetry. Moreover, based on inhibition of choline acetyl transferase (ChAT), the studied neurotoxic effect, we performed benchmark dose (BMD) modeling with a benchmark response (BMR) of one standard deviation and selected the upper BMD for in the *in vivo* dose, for prediction of neurotoxicity in humans. To assess the predictivity of the QIVIVE approach and the used *in vitro* cellular model, the result was compared to doses associated with neurotoxicity in patients, as reported in the literature (Smith et al., 1986; Kerin et al., 1989; Orr and Ahlskog, 2009).

#### 2.2.2 Model Structure and Physiological Parameter Values

A published rat kinetic model (Lu et al., 2016) for AMI was parameterized for an adult human by incorporating standard human physiological parameters including cardiac output, organ weights, and blood flows (Brown et al., 1997). The model consisted of 10 tissue compartments including the target organs relevant for AMI adverse effects and the arterial and venous blood supply. The circulation system was closed *via* the lung and the heart. Elimination was by metabolism in the liver to give MDEA as a primary metabolite, while other tissue compartments, including the brain, were regarded as non-metabolizing. Indeed, although some metabolic activity can be assumed at the brain level as witnessed by the formation of MDEA in the *in vitro* biokinetic study, it amounted only to 2.5–3% compared to 50–60% seen in liver cells. It was thus deemed not to contribute to the overall body clearance. Drug input was modeled by both intravenous (i.v.) and oral routes. The model output was validated by comparing the model outcome with kinetic data in human clinical studies after i.v. and after oral administration of AMI.

#### 2.2.3 Drug-Specific Parameters

Amiodarone is a poorly soluble, lipophilic compound (log *P*_O/W_, 7.57) (Avdeef et al., 1998) with high protein-binding capacity (96%–99%) and shows extensive tissue distribution. Hepatic clearance to MDEA was parameterized with *V*_max_ (4.12 nmol/mg/h) and *K*_m_ (38.85 μmol/L) from an *in vitro* study in human liver microsomes (Trivier et al., 1993) and was scaled to total organ clearance to give an intrinsic hepatic clearance of 5.07 L/h using physiological scaling factors, as described previously (Barter et al., 2007). This value was close to the lower end of reported values in the literature for AMI *in vivo* clearance, that is, 8–46 L/h (Riva et al., 1982; Latini et al., 1984). The intrinsic hepatic clearance to give other metabolites accounting for 10% of total clearance was taken from Chen et al. (2015) and was, likewise, scaled to give a clearance of 0.096 L/h. Protein binding was tested in a range of 96–99.9%. For the oral model, a first-order absorption rate constant (k_a_) of 0.323 h^−1^ and a fraction absorbed (f_a_) of 0.3 were taken from Kannan et al. (1982). Relevant model input parameters are summarized in Table 1.

#### 2.2.4 Program Assumptions

Drug distribution into the liver was modeled by permeability rate-limited kinetics, where vascular and extravascular spaces are separated by a diffusional barrier which becomes the limiting process. The permeability–surface area product (PS × tissue) and the tissue-specific unbound fraction of AMI (fu) described the transport of molecules between the two sub-compartments. The rat parameters were used to describe the AMI tissue-partitioning properties in the human model, which could be justified by the similarity of membrane and tissue composition in terms of water, proteins, and lipoproteins. We used Berkeley Madonna software for simulation (version 8.3.18).

Evaluation of the i.v. h-PBK model was performed by comparing the simulation results with the plasma concentration–time profile observed in patients with cardiac arrhythmias after a single i.v. dose of 400 mg AMI (Andreasen et al., 1981). Similarly, the output of the oral model was compared with plasma concentration–time data after oral intake of AMI in humans (Kannan et al., 1982). We also varied the fu of AMI in the range of 0.06–0.001 during the evaluation while maintaining the other model parameters unchanged. Alternatively, AMI clearance was calculated by using *V*_max_ and *K*_m_ from the *in vitro* biokinetic study and scaled to the total organ clearance.

The program code is available from Lu et al. (2016).

BMD modeling of the measured neurotoxic parameter was performed using the R package PROAST (version 67.0).

#### 2.2.5 Dose Finding by QIVIVE

Liver model: we modeled the *in vivo* doses starting from the *in vitro* actual measured concentrations in the medium and in the cell lysate. Performing QIVIVE, we used both the scaled metabolic clearance parameters (*V*_max_ and *K*_m_) from human liver microsomes and the metabolic parameters taken from the *in vitro* biokinetic data. We also set the protein binding at 0% for the modeling, reflecting the conditions used in the *in vitro* study due to the use of a protein-free *in vitro* culture medium. In those conditions, the medium could not bind to AMI in contrast to the *in vivo* situation, where AMI has a high affinity for plasma proteins. In an iterative process, we increased the dose given into the human system, and simulations for internal concentrations in plasma and liver cells were performed. An optimized dose was obtained by applying SSQ minimization between the predicted and the *in vitro* experimental concentrations.

Brain model: we used a validated h-PBK model to directly perform reverse dosimetry (Algharably et al., 2019). Here, we relied primarily on the calculated AUC_0-24_ for AMI concentrations measured in the brain cell lysate on day 14 as a target metric to perform QIVIVE for the two nominal concentrations, that is, 1.25 and 2.5 μM. We iterated the doses in steps of 0.01 mg AMI given to the human system and selected accordingly an optimized dose which had the smallest difference between the *in silico* predicted AUC and the *in vitro* observed value. Using the toxic effects measured in the *in vitro* study but related to nominal concentration, we were able to translate the *in vitro* concentration–response data into *in vivo* dose–response curve in humans for ChAT activity, for which we performed BMD modeling. The benchmark response (BMR) was chosen as an effect size of one standard deviation (SD) of the background response. The upper bound for BMD (BMDU) was selected to predict a dose able to elicit adverse effects on the CNS, which will be subsequently used for comparisons with doses associated with neurotoxicity from clinical studies.

### 2.3 Case 3 (Chlorpyrifos, Algharably et al., in Preparation)

#### 2.3.1 Scenario

Data from an *in vitro* biokinetic and toxicodynamic study (Di Consiglio et al., 2020) were the basis to perform a human QIVIVE. In this study, the time courses of CPF and its toxic metabolite CPF-oxon (CPFO) concentrations were measured in the medium and in the cell lysate of human-induced pluripotent stem cell-derived neural stem cells (hiPSC-NSCs) undergoing differentiation and repeatedly exposed for 14 days to CPF. The selected *in vitro* concentration (nominal: 21 μM) corresponded to IC5 (concentration causing 5% reduction of viability). The measured concentration in the medium was considered to represent the plasma/blood concentration, and the concentration in the cells was considered to represent the one bioavailable at the target organ. The Open Systems Pharmacology (OSP) suite was used to build the model of a pregnant woman at the end of the first trimester, also including the fetus as a tissue representing the target organ for toxicity. This model has been used to perform simulation of concentrations in the fetus enabling comparing concentrations measured in the cells *in vitro* with concentrations in the fetus.

In a second step, we evaluated the *in vitro* effects related to developmental neurotoxicity and measured in hiPSC-NSCs, after 14 days of repeated treatment. The differentiation status of the cell cultures characterized by neurogenesis, gliogenesis, neurite outgrowth, and synaptogenesis would correspond to the development of a human fetus at the end of the first trimester (Douet et al., 2014). The *in vitro* concentration—effect data were used to perform a BMD analysis to determine the BMDU with a BMR of one SD of the measurements in the control. The *in vitro* BMDU was converted into *in vivo* concentration in the human fetus by using the established kinetic model of the pregnant mother and her fetus.

We then assessed data from human epidemiological studies in which both the effects measured in the offspring of pregnant women and the blood concentration values in the mothers were available (Rauh et al., 2006; Silver et al., 2017; Chiu et al., 2021). Using the kinetic model, we simulated the concentrations in the fetus, corresponding to the concentrations measured in the mothers taken from the publications, and compared them with the BMDU, converted from nominal to actual concentrations, for the *in vitro* observed effects. In this model, our aim was also to investigate possible MoA for the developmental neurotoxicity elicited by CPF other than the well-known inhibition of acetylcholinesterase (AChE).

#### 2.3.2 Model Structure and Physiological Parameter Values

We used the OSP suite to build a model for a pregnant woman and her fetus. A reference h-PBK model for CPF for an adult non-pregnant woman was first developed in PK-Sim^®^ incorporating the PK-Sim^®^ standard model structure comprising 18 compartments (Krauss et al., 2012) and then was extended to a pregnant woman in MoBi^®^. Some of the physiological parameters were retrieved from the literature and by expert opinion. Drug input was modeled by the oral route as single-dose exposure, whereas for elimination only the hepatic clearance was considered, using CYP-specific *V*_max_ and *K*_m_ from an *in vitro* study with human hepatic microsomes (Zhao et al., 2019). In this model, the RBC/plasma ratio and blood–brain barrier that are both relevant to CPF were calculated as partition coefficients according to the different tissue/plasma partition coefficient algorithms implemented in the software (Schmitt 2008b).

#### 2.3.3 Drug-Specific Parameters

Physicochemical properties of CPF (molecular weight, 350.57 g/mol; log *P*_O/W_, 4.96; and solubility, 1.4 mg/L) retrieved from PubChem and a non-protein-bound fraction of 0.03 (Timchalk et al., 2002) were implemented in the molecule building unit. For parameterizing absorption, we used a specific intestinal permeability for CPF taken from an *in vitro* study that assessed the intestinal uptake of CPF using the single-pass intestinal perfusion method in rats (Cook and Shenoy, 2003). Relevant model input parameters are summarized in Table 1.

#### 2.3.4 Program Assumptions

The software used for PBK modeling was PK-Sim^®^. The structure of the model was similar to that used in the other examples where compartments represent organs/tissues, and the different organs were connected by the arterial blood flow and venous blood flow. The compartments are composed of sub-compartments, representing the vascular space which is sub-divided into plasma and (red) blood cells, the non-vascular space which is sub-divided into interstitial space and cellular space. The system of time-dependent differential equations is solved numerically. PBK simulations deliver concentration–time courses of the compound in the various compartments, which are addressed in the equations. It is explicitly said, in the information package, that PK-Sim^®^ is designed for use by non-modeling experts and that it only allows for minor structural model modifications. For further details, see program description (https://www.open-systems-pharmacology.org/).

#### 2.3.5 Comparison of the *In Vitro* Effect Concentrations With *In Vivo* Effect Concentrations in the Fetus

Among the several publications in the open literature in which isolated single plasma or cord blood concentrations in epidemiological studies were measured in pregnant women, although with unknown “external” exposure to CPF, we selected the studies in which the offspring showed adverse neurodevelopmental effects later in life (Rauh et al., 2006; Silver et al., 2017; Chiu et al., 2021). We compared the *in vitro* upper concentration (BMCU) converted from nominal to actual concentration from the BMD modeling for the *in vitro* observed effects with the modeled concentrations in the fetus. This allowed assessing the suitability of the selected *in vitro* experimental system and the endpoints tested to predict the neurodevelopmental effects in quantitative terms.

## 3 Results

### 3.1 Case 1 (IBU)

#### 3.1.1 Kinetic Model Evaluation

When using the tissue/blood partitioning coefficient calculated by the algorithm of Schmitt (Schmitt, 2008a; Schmitt, 2008b) of 3.01, the non-protein-bound (fu) IBU of 0.01, and the *in vivo* clearance of 0.06 kg bw (L/h) (corresponding to 79 ml/min), the simulated concentration–time profile in blood and the *in vivo* measured one matched very well, confirming the validity of the h-PBK model and the applied parameters. In this respect, model predictions were checked visually and the differences between the predicted data compared to observed data were within an order of magnitude, which is considered acceptable in previous publications (Abdullah et al., 2016; Pletz et al., 2020; Punt et al., 2021). In contrast, when using the ratio of the *in vitro* concentration in the hepatic cell lysate and in the medium to calculate the partition coefficient liver tissue/blood, which was 11.1, the simulated concentration–time profile did not fit well with the experimental data. However, comparing the SSQs for the distance between experimental and simulated data demonstrated clearly that the simulation with the calculated liver tissue/blood partitioning coefficient fitted the experimental data better.

The major difference about the *in vitro* and the *in vivo* situation was the low protein content of the culture medium (Williams’ E medium and the Geltrex™) used in the *in vitro* experiment. Hence, we evaluated the influence of the non-protein-bound fraction (fu) on the *in silico* predicted liver/blood partition coefficient by using the *in silico* prediction algorithm by Schmitt (2008a) and Schmitt (2008b). We found out that with increasing fu, the liver/blood partition coefficient increased. Hence, because the free fraction of IBU in the *in vitro* medium was much higher than that in the blood *in vivo*, a higher *in vitro* partitioning into the human hepatic cells did result*.*

Because the clearance values from the *in vitro* and *in vivo* studies were only differing about 10%, the results of the simulations did not differ when using the same partition coefficient, calculated by the Schmitt algorithm, for simulation.

#### 3.1.2 Dose Finding

**FIGURE 1:**
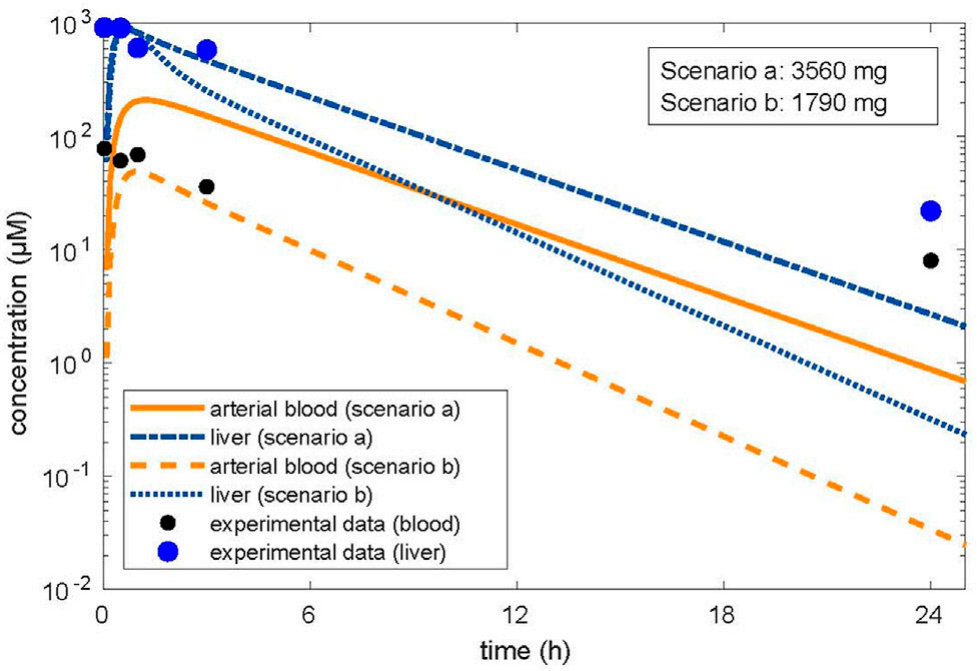
Optimized dose in reverse dosimetry with optimization for concentrations in the target organ of toxicity (liver). Condition/scenario a: hepatic tissue: blood partition coefficient of 3.01 calculated by the algorithm of Schmitt, (2008a; Schmitt, 2008b). Condition/scenario b: hepatic tissue: blood partition coefficient of 11.1 derived from the *in vitro* data (concentration in human hepatic cells/concentration in the supernatant) calculated from the *in vitro* concentration–time data (Truisi et al., 2015). Orange color: concentrations in arterial blood; blue color: concentration in the liver. Orange line and dashed-dotted blue line: condition (a); orange dashed line and blue dotted line: condition (b). Modeling using the hepatic tissue: blood partition coefficient calculated by using the *in vitro* measured concentration in the cell lysate and the concentration in the medium fits the *in vitro* data well; however, the resulting dose related to beginning liver toxicity is within the therapeutic range. Modeling using the hepatic tissue: blood partition coefficient calculated by the algorithm (Schmitt, 2008a; Schmitt, 2008b) did not well describe the concentration in the medium when optimized for the liver concentration; however, the resulting dose related to beginning liver toxicity is in accordance with the clinical observations.

In the first approach, the in vitro clearance and the in vitro partition coefficient (cell lysate: medium) of 11.1 were used in the simulation, in order to estimate whether *in vivo* concentrations in humans in plasma and the liver were similar to the *in vitro* experiment in the medium and cell lysate. The concentration–time profile up to 3 h was in line with the experimental *in vitro* data. In the second step, we optimized the dose based on the concentration in the cell lysate by using the same parameters as in the validation step, that is, the tissue/blood partitioning coefficient calculated by the algorithm of Schmitt, fu = 0.01 and the *in vivo* clearance. A lower dose of 1,790 mg resulted from the first step than from the second step, which resulted in a dose of 3,560 mg (Figure 1). We assumed that both doses were predicting low oral toxicity because the *in vitro* concentration used corresponded to TC10. The difference between the two doses is explained because using the higher *in vitro* partition coefficient for liver/blood in the modeling results in higher predicted *in vivo* liver concentrations than in the calculations using the predicted *in silico* partition coefficient for liver/blood. Thus, the *in vitro* prediction for the dose is 1.54-fold lower than the prediction with the calculated partition coefficient which, however, is in agreement with human post-mortem partitioning between the liver and blood (Table 2).

**TABLE 2:** Case 1—IBU-: Relevant data and results.

Parameter	*In vitro* data	Human data
fu	Not measured, protein-free medium	0.01
Partition coefficient	11.1	2.7^a^
		3.01^b^
Clearance (L/h)	3.6	4.2
Simulated daily dose (mg) with low toxicity	1,790^c^	3,560^d^
Therapeutic daily dose (mg)		up to 2,400
Dose (mg) with severe intoxication requiring liver transplantation		9,000

fu, fraction unbound.

a Post-mortem data (Kunsman and Rohrig, 1993).

b Calculated according to (Schmitt, 2008a; Schmitt, 2008b).

c Optimized dose in reverse dosimetry with optimization for concentrations both in the medium and in the human hepatic cells using the *in vitro* parameters for hepatic tissue/blood partition coefficient and clearance.

d Optimized dose in reverse dosimetry with optimization for concentrations in the human hepatic cells (target of toxicity) using the *in vivo* parameters for hepatic tissue/blood partition coefficient and clearance.

#### 3.1.3 Comparison of the Outcome of Modeling With Clinical Data

A MoA for hepatotoxicity is not established, and data are not available to allow describing a MoA according to Boobis et al. (2008). However, data on therapeutic doses and case reports on liver toxicity allow in assessing the relevance of the predicted doses. The daily therapeutic dose of IBU is up to 2,400 mg, indicating that frank toxicity is not expected at 1,790 mg, the QIVIVE dose, resulting from simulations with the partition coefficient of 11.1, calculated from *in vitro* measured data (Table 2). In the literature, two cases were reported on suicidal attempts with detailed information on the IBU doses ingested. In one case, a dose of 9,600 mg (Laurent et al., 2000) led to severe liver toxicity, requiring liver transplantation. In the second case, acute liver toxicity occurred, following the ingestion of 20,000 mg IBU (Lee and Finkler, 1986). Hence, the dose of 1,790 mg is within the therapeutic range and too low to predict liver toxicity *in vivo* in humans. The dose of 3,560 mg is one-third of the dose in the case reported by Laurent et al. (2000) with severe liver toxicity and might be considered as an estimate for a low toxic dose (Table 2). This dose was simulated using an *in silico* calculated partition coefficient for the liver of 3.01, which was in accordance with the partition coefficient for liver/blood of 2.7, calculated from post-mortem data in a human subject having committed suicide with IBU (Kunsman and Rohrig, 1993).

### 3.2 Case 2 (AMI)

#### 3.2.1 Comparison of the Outcome of Modeling With Clinical Data

**FIGURE 2:**
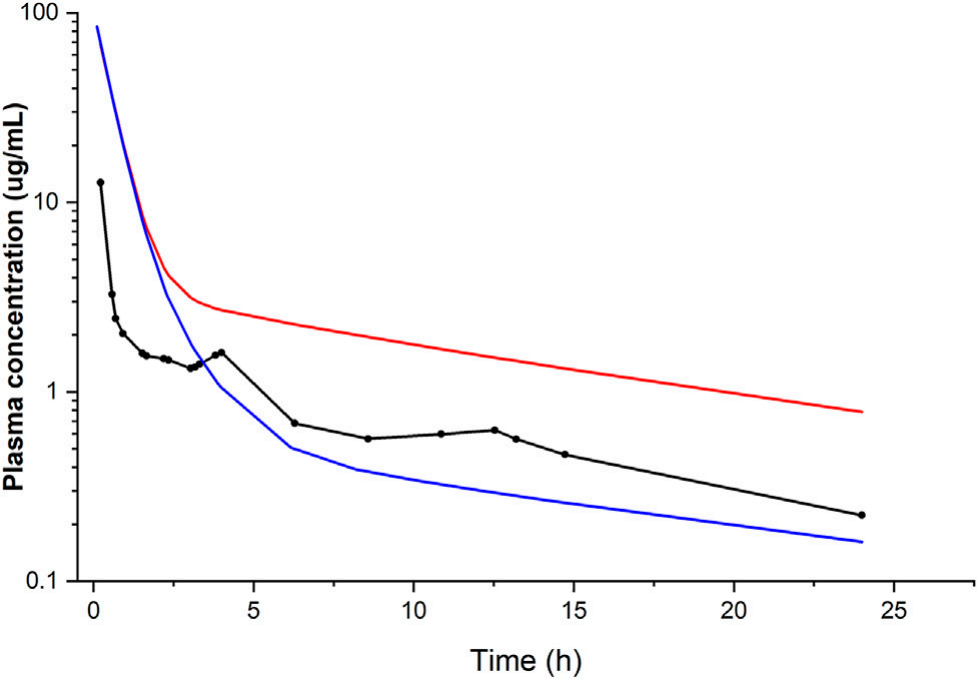
Simulated (lines) and observed (points) plasma concentration–time profiles of amiodarone following i.v. administration of 400 mg. Conditions: fu of 0.06 and hepatic clearance calculated from *in vitro* data (Trivier et al., 1993) (blue line) or alternatively obtained from *in vitro* data (Pomponio et al., 2015a) (red line), compared with the average plasma concentration–time data of seven patients with cardiac arrhythmias (Andreasen et al., 1981).

Liver model: when using the clearance value based on the *in vitro* data from human liver microsomes (Trivier et al., 1993), the simulated plasma concentration–time profile was in good agreement with that observed in an *in vivo* study after i.v. (Andreasen et al., 1981) as well as oral administration (Kannan et al., 1982), thus validating our h-PBK model. The output of the model was sensitive to variations in protein binding, which was the most important variable affecting the goodness of fit. In contrast, when the clearance was based on the metabolic parameters obtained from the *in vitro* biokinetic study in PHH (Pomponio et al., 2015a), corresponding to an intrinsic hepatic clearance of 0.002 L/h, the predicted *in vivo* plasma concentration–time profile was not comparable with that observed in the *in vivo* clinical studies (Figure 2). This was attributed to the extremely low clearance observed in the PHH culture. We explained the low *in vitro* clearance by the fact that the concentration of the metabolite MDEA, known for its inhibitory effect (*K*i 12.1 μM) on CYP3A4, the relevant CYP enzyme for AMI metabolism, accumulated inside the cells (Pomponio et al., 2015a) and slowed down the rate of AMI metabolism.

Brain model: the original model developed and validated in the rat was ascertained to predict internal concentrations in the brain by comparing the model predictions to published studies with *in vivo* tissue concentrations in the rat before we used the parameterized human model for QIVIVE. A MoA for neurotoxicity is not established, and data are not available to allow building a MoA according to Boobis et al. (2008). However, data from clinical studies reporting on neurotoxicity allow in assessing the validity of the predicted doses.

#### 3.2.2 Dose Finding

Liver model: when the h-PBK model was parameterized with *in vitro* validated parameters (clearance 5.068 L/h; fu 0.06), we noticed large discrepancies between the simulated concentration–time profile in the plasma and the liver and the *in vitro* data in both the medium and hepatocytes, respectively.

Notably, the predicted concentrations, particularly in the liver, mismatched with those observed experimentally *in vitro* showing initially high concentrations and then dropping to lower values, thus rendering dose predictions by QIVIVE difficult to perform. Accordingly, an *in vivo* dose of 7 mg/kg bw could be obtained for the medium whereas an *in vivo* dose at least 10-fold higher was necessary to reach the intracellular liver concentrations. This could be due to an initial higher *in vitro* intracellular drug concentration due to lower protein binding, resulting in a higher free fraction of AMI, thus available for and partitioning into hepatocytes. Model prediction could only be improved when we parameterized the model with metabolic parameters from the same *in vitro* study (Pomponio et al., 2015a), thus with an extremely low clearance (0.002 L/h) and when protein binding was not accounted for. In these “unphysiological” conditions, that is, 0% protein binding; fu = 1, the obtained profiles were in agreement with *in vitro* experimental data.

Brain model: the AUCs in the brain cell culture resulting after 14-day repeated dosing of the AMI nominal concentrations (1.25 and 2.5 μM) were 1.00 and 1.99 μg h/ml, respectively. The corresponding doses calculated by QIVIVE were 3.83 and 7.68 mg/kg, respectively. Notably, dose predictions based on using the *C*_max_ as a kinetic metric were also comparable, amounting to 3.76 and 7.12 mg/kg, respectively. The selection of BMDU enabled us to model a dose that is associated with adverse effects *in vivo* (Supplementary Figure S1). Consequently, an i.v.-modeled BMDU of 5.28 and 5.09 mg/kg based on the AUC and *C*_max_ approaches, respectively, were obtained. Applying a bioavailability of 65%, taken from the literature (Pourbaix et al., 1985), these corresponded to oral doses of 593 and 571.6 mg for a 70 kg person using AUC or *C*_max_ as the respective relevant metric. In contrast, the i.v. BMDU based on the nominal concentration was found to be 0.058 mg/kg, which gave an oral dose of 6.5 mg for a 70 kg adult human. However, the nominal concentrations should be corrected since they are to be regarded as AMI free fraction. Therefore, after adjusting by a correction factor for unbound fraction [multiplying by 100/unbound fraction (fu = 0.06)], it resulted in an oral dose of 10,833.3 mg.

#### 3.2.3 Validation of the Effect Outcome by Clinical Data

Liver model: we did not compare the results of QIVIVE since the relevance of the *in vitro* system for undertaking dose predictions was questionable, considering the stark differences in protein binding and drug clearance capacity between the *in vitro* system and the *in vivo* situation. This has special importance for highly protein-bound drugs such as AMI.

Brain model: the obtained BMDU and subsequently the total oral doses predicted to cause neurotoxicity were concordant with AMI doses reported to be associated with neurological adverse effects in clinical studies. As such, oral doses of 400–500 mg were associated with neurotoxicity with variable symptoms, including muscle weakness, fatigue, tremor, ataxia, peripheral neuropathy, and cognitive impairment/encephalopathy (Smith et al., 1986; Kerin et al., 1989; Orr and Ahlskog, 2009). In contrast, dose prediction based on the nominal concentrations resulted in an oral BMDU of 10,833.3 mg that exceeded the clinical doses which are in use and already eliciting adverse effects to such an extent that they would be highly toxic if not lethal. This highlights the inadequacy of using the nominal concentrations to predict *in vivo* toxicity.

Table 3 captures the most relevant data and results of this case.

**TABLE 3:** Case 2—AMI - : Relevant data and results.

Parameter	*In vitro* data	*In vivo* human data
**A. Kinetic**		
Clearance (L/h)	0.002	5.07
Fraction unbound	not measured, protein-free medium	0.06
Agreement with *in vivo* concentration–time profile	poor	good
**B. Dynamic**		
QIVIVE dose (mg)		Doses in patients with neurological side effects (mg)
BMDU based on measured *in vitro* intracellular AUC_0-24_	593	400–500
BMDU based on nominal concentration	10,833.3	

### 3.3 Case 3 (CPF)

#### 3.3.1 Kinetic Model Evaluation

**FIGURE 3:**
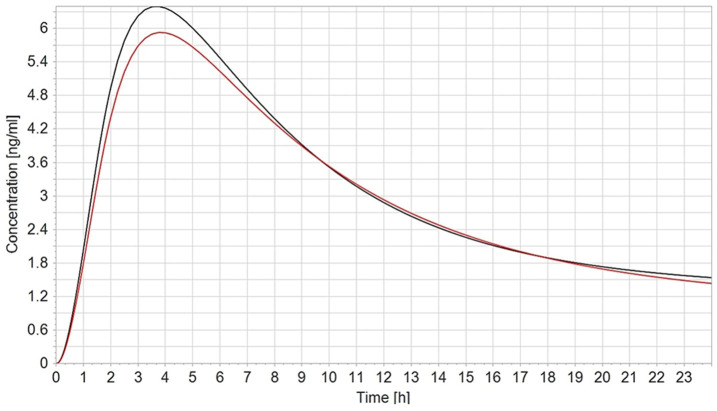
Simulated plasma CPF concentration–time profile in non-pregnant (black line) vs. pregnant (red line) population (*n* = 100) after a single oral dose of 2 mg/kg.

To validate the PBK model, the predicted plasma concentrations of CPF were compared with the reported *in vivo* data. In humans, robust data on the kinetics of CPF, the parent compound, are scarce. In a controlled study by the US EPA (Brzak, 2000) applying oral doses of 1–2 mg/kg bw in male and female volunteers, serum CPF detected in some of the subjects ranged from 1.1 to 5.6 ng/g and 1.3–18 ng/g in the 1.0 mg/kg and in the 2.0 mg/kg, respectively. The model-predicted plasma concentrations of CPF with 1 and 2 mg/kg oral doses in female and male adult humans were in good agreement with concentration–time data observed in the human studies. On the other hand, no kinetic studies in pregnant women exposed to defined doses of CPF are reported in the literature. Only the plasma concentration in pregnant women from biomonitoring studies was available. Hence, validation of the pregnant model was not feasible. However, the observed small differences to the non-pregnant concentration–time profile are explained by the physiological changes in pregnancy, in accordance with the changed physiological parameters which we implemented (Figure 3).

#### 3.3.2 Comparison of the *In Vitro* Effect Concentrations With *In Vivo* Effect Concentrations in Pregnant Women

The tested nominal concentrations were between 18.45 and 37.1 μM (Di Consiglio et al., 2020). We performed BMD modeling and assessed the results by considering the intervals between the BMDL and the BMDU and the distance of the BMDL from the lowest experimental concentration. In only two measured effects, that is, the number of neurites/neuron and those of synapses, the intervals were acceptable, and the distance was small. We selected the BMDU as the relevant metric to account for the fact that we would compare an adverse effect-inducing *in vivo* concentration with the corresponding *in vitro* concentration. Hence, the BMDU is the relevant concentration. For the endpoint neurites/neuron the nominal BMDU was 29.5 μM (corresponding to 10.3 μg/ml), converted to an actual intracellular concentration of 11.7 μg/ml, and for the endpoint synapses, the nominal BMDU was 25.7* *μM (corresponding to 9.0 μg/ml), converted to an actual intracellular concentration of 10.2 μg/ml (Table 4).

**TABLE 4:** Case 3—CPF-: Relevant data and results.

Epidemiological study with neurodevelopmental effects in infants from mothers exposed during pregnancy	Concentration in blood measured in exposed pregnant women (ng/ml)	Simulated concentration in the fetus (ng/ml)	BMDU from the *in vitro* study for two neurodevelopmental-related endpoints (ng/ml)
Silver et al. (2017)	0.56–7.33	0.82–10.7	10,200 and 11,700
Chiu et al. (2021)	0.13–5.29	0.19–7.75	
Rauh et al. (2006)	0.011	0.02	

In Silver et al. (2017), the CPF blood concentrations in the exposed pregnant women were between 0.56 and 7.33 ng/ml. In the infants, the motor function was assessed at 6 weeks and 9 months, and the scores were significantly lower for exposed vs. unexposed infants. In Chiu et al. (2021), the CPF blood concentrations in the exposed pregnant women ranged between 0.13 and 5.29 ng/ml, and a concentration-dependent statistically significant poorer performance in the cognitive and language domains was observed at the age of 2 years. The lowest blood concentrations related to adverse neurodevelopmental effects were reported by Rauh et al. (2006), where attention-deficit/hyperactivity disorder problems were observed in children; differences comparing high (above 0.011 ng/ml) vs. low exposed children were statistically significant. When we simulated the plasma CPF concentration in the fetus as the mother was exposed to the same dose that we estimated from those studies by reverse dosimetry (data not shown), the resulting simulated concentrations in the fetus were 0.82–10.7 ng/ml (Silver et al., 2017), 0.19–7.75 ng/ml (Chiu et al., 2021), and 0.02 ng/ml (Rauh et al., 2006) (Table 4). As we compared the blood concentrations in epidemiological studies showing neurodevelopmental effects with the BMDU concentrations of *in vitro* studies showing subtle effects related to neurodevelopment, a difference of several orders of magnitude was evident. These findings are interesting since the model and the measured parameters are considered a fair representation of the developing brain (Bal-Price et al., 2018; Pistollato et al., 2020).

## 4. Discussion

### 4.1 Toxicokinetic Aspects

#### 4.1.1 Protein Binding

In two of the reported examples with highly protein-bound drugs (i.e., IBU and AMI), it is obvious that the protein content in the media used for the *in vitro* cell culture is an important determinant for the dose/concentration, which causes a toxic effect. Here, the difference in the protein content in plasma and in the media is remarkable. For IBU, the influence of a lower protein binding in plasma/medium on the partition coefficient was demonstrated with the theoretical calculations using the Schmitt algorithm, resulting in an increasing tissue/cellular concentration with decreasing protein binding. Recently, the influence of plasma protein binding was addressed not with respect to implications for the partitioning into the tissue (as we did) but with respect to influencing the QIVIVE of hepatic clearance, in particular, considering plasma protein-mediated uptake (Bteich et al., 2019; Francis et al., 2021; Poulin and Haddad, 2021). In the example with IBU, although being a highly protein-bound drug, the partitioning into the tissue is strongly affected, we observed only a 14% lower predicted *in vitro* clearance than *in vivo*, which is within the normal variability. Hence, in this case the indicated influence of plasma protein on the hepatic clearance seemed not to have an impact.

#### 4.1.2 *In Vitro* Clearance

In the example of AMI, the *in vitro* clearance differed from the *in vivo* clearance despite the use of PHH as a relevant human cellular model and the measurement of both AMI and its metabolite MDEA. The most probable explanation is the fact that the metabolite of AMI is known to inhibit the metabolism of the parent compound. A similar case was observed in the study of Schug et al. (2013). The lower *in vitro* clearance compared to the *in vivo* clearance resulted in effects in the *in vitro* primary rat hepatocyte culture that were not observed in the liver of a corresponding *in vivo* study in rats, most probably because of prolonged *in vitro* exposure of the hepatocytes (Schug et al., 2013). This example shows the interplay between *in vitro* kinetics and the related dynamics.

#### 4.1.3 Nominal Concentrations

*In vitro* benchmark concentrations, like EC20 values, are routinely derived from dose-response curves, which are based on a range of nominal treatment concentrations. When these nominal parameters are directly applied to a QIVIVE, the approach inherits a relatively high amount of uncertainty. Indeed, the nominal concentrations do not account for the distribution of compounds within the *in vitro* test system, which determines the true free concentration of the tested compound, responsible for the induction of the measured toxicological effect. This is mainly important in the case of compounds with high-binding affinity to serum constituents (Groothuis et al., 2019). Nominal concentrations are nonetheless in use for screening purposes, for example, in the US EPA ToxCast/ExpoCast project (Wetmore et al., 2015; Wambaugh et al., 2019). In the example of AMI, it was demonstrated that using the nominal concentration for BMD modeling would result in a predicted dose for toxicity several folds higher than the dose calculated when using the intracellular measured concentration. In this case, the outcome of the *in vivo* human dose–response modeling resulted in a BMDU of 593 mg based on AUC metric and 572 mg based on *C*_max_ metric, the doses being in excellent agreement with the results of clinical studies, in which doses of 400–600 mg were associated with the signs of neurotoxicity. The dose prediction based on nominal concentrations resulted in a BMDU of 10,833.3 mg, which is clearly an extreme over-prediction. Measurements of intracellular *in vitro* concentrations as well as in the media are generally workload intensive; however, *in silico* distribution models have been developed to estimate the *in vitro* free concentration and *in vitro* cell–associated concentrations. An extensive review has been recently published, discussing the predictivity and potential application domains of the different approaches published so far (Proença et al., 2021). However, the application of *in silico* models becomes challenging when specific *in vitro* conditions apply, for example, short acute exposure or exposure to rapidly metabolized or slowly permeable chemicals.

### 4.2 Toxicodynamic Effects

#### 4.2.1 Model Capability to Predict *In Vivo* Adverse Effects

Concerning the results of toxicodynamics, we were able to validate the predicted outcomes in all three cases by the available effect data in humans. There are not many examples in the literature in which predicted *in vivo* doses in humans are compared with therapeutically used doses or doses which elicit adverse side effects in humans. Kasteel et al. (2021) predicted therapeutic doses of baclofen to treat spasticity, with the *in vitro* IC50 as the reference point. Compared with the therapeutically recommended doses, they were successful in predicting orally as well as intravenously administered doses, however not intrathecal doses, most probably because a more complex structural model for drug distribution would be necessary to mirror the therapeutic scenario. In two of the three cases we presented here, IBU and AMI, the prediction from the *in vitro* effect matched well with the clinical data on hepatic impairment (IBU) and on neurological side effects (AMI). However, in case of CPF, the *in vitro* prediction does not match with the exposures measured in pregnant women and causing neurodevelopmental toxicity in their offspring using the previously mentioned *in vitro* endpoints, that is, synapses and neurite growth. For the quantitative prediction of the *in vivo* dose which is eliciting adverse effects in humans, three points are relevant. First, it is important to be sure that the cell culture in which the effect was measured is biologically similar to the *in vivo* target tissue and is reacting with the same sensitivity. For IBU, the *in vitro* culture was PHH, and they were more sensitive than primary rat hepatocytes or other cells with different origin, for example, from skin [Table 2 in Mielke et al. (2017)], making the PHH model relevant for the primary target of IBU toxicity, the liver, in humans. For testing AMI, the rat brain cell culture was used, and the dose predictions matched well, indicating that in this case, the cellular model used was predictive. In the case of CPF, the cellular model used was a culture of human-induced pluripotent stem cell-derived neural stem cells undergoing differentiation. This model is considered as a fair representation of the developing brain (Bal-Price et al., 2018; Pistollato et al., 2020). Second, it is important to know whether the *in vitro* effect measured is related to the MoA *in vivo*. For IBU, the *in vitro* effect is cytotoxic, which is an excellent model for direct liver toxicity observed in cases of suicidal attempts. The *in vitro* effect of AMI was inhibition of choline acetyl transferase. Data in the literature make plausible that inhibition of choline acetyl transferase might play a role in the MoA for the observed neurological side effects (Algharably et al., 2021). In case of CPF, the quantitative *in vitro* prediction does not match with the exposures which were measured in pregnant women and which caused neurodevelopmental effects in their offspring’s later life. We used epidemiological studies which reported neurodevelopmental effects in children after CPF *in utero* exposure, using blood concentrations from biomonitoring data in the mothers and applying reverse dosimetry, thus predicting the external exposure dose, which was then used as input to the model to further predict the would-be resulting fetal concentrations. In this approach, much lower concentrations were simulated in the fetuses than the concentrations, which showed *in vitro* effects in neuronal cells. We applied this approach due to lack of data on pregnant women.

Concerning the MoA of CPF toxicity, although acetylcholine esterase (AChE) inhibition has been an established mechanism for toxicity, it has been shown that other mechanisms might also play a role in neurodevelopmental effects. For example, it has been shown that non-cholinesterase-depending mechanisms induced by CPF can alter synaptogenesis, neuronal network formation, and BDNF signaling in differentiating PC12 cells *in vitro* but also in young rats *in vivo*. It is also described that neurite outgrowth is inhibited by CPF in the PC12 cells and in primary cultures of embryonic rat sympathetic neurons. These results were the reasons why we used the *in vitro* test battery established for neurodevelopmental neurotoxicity, as described in Di Consiglio et al. (2020). The aim of this study was indeed to evaluate the quantitative predictive value for developmental neurotoxicity of the *in vitro* model, which is assumed to be promising in this respect (Pistollato et al., 2020).

### 4.3 Modeling Platforms

For the purpose of PBK modeling, a number of generic modeling platforms (Berkeley Madonna, MATLAB, R, or almost any programming language) and also a number of specialized PBK modeling platforms exist. Concerning the latter, open-source modeling platforms [IndusChemFate (Cefic LRI), high-throughput toxicokinetics (httk)-R package, MEGEN-RVis, PLETHEM, MERLIN-Expo, and PK-Sim^®^] and license-based platforms (GastroPlus and SimCyp) can be used, requiring varying degrees of expertise in PBK. We gained experience with some of these platforms. When using a generic platform, all equations and all parameters are part of the code. This is more labor intensive at the beginning; however, it gives flexibility to adapt to the model structure and its complexity to the situation, taking into account the relevant physiological factors. In addition, all relevant information is contained explicitly in the code. This allows for maximum transparency and completeness of the model. We had used this approach for case 1 and case 2 with MATLAB and Berkley Madonna, respectively. When using specialized software, there are predefined modules for anatomical entities or physiological processes (implicitly implying parameters and equations). This is convenient, but transparency might become a problem and it might be difficult to implement specific physiological situations which require changes in the standard parameters. We showed this with IBU fitting, comparing the difference in modeling outcomes using the same input parameters in PK-Sim^®^ and in our original model in MATLAB (Supplementary Figure S2). Although the model in PK-Sim^®^ was convenient to build, the model flexibility was lower when the model outcome was fitted to the available clinical human study; therefore, dose optimization by reverse dosimetry was not easy to perform. Notwithstanding, the simulated plasma concentration was in the same order of magnitude as those predicted from the generic model in MATLAB.

### 4.4 Lessons learned

In summary, our work showed the applicability of QIVIVE and that it can provide reliable results, when compared against *in vivo* data. When performing reverse dosimetry, several points have to be taken into consideration.

All modeling approaches can be applied, and we experienced their pros and cons. In the three cases, we were successful in predicting the *in vivo* concentration–time profile and for CPF at least for non-pregnant women, thus establishing confidence in the predictivity of the kinetic model and the selected physiological and substance specific data. Furthermore, the concentration–time profile in the case of CPF for pregnant women was in accordance with the expected differences due to pregnancy-related changes in physiology with lower *C*_max_ due to a higher volume of distribution. The concentration time profile in the case of CPF for pregnant women was in accordance with the expected differences due to pregnant-related changes in physiology. For meaningful quantitative effect extrapolation from *in vitro* to *in vivo* conditions, it is essential to be aware of the potential impact of the selected *in vitro* conditions, making differences with the *in vivo* situation (e.g., the use of serum-free medium). In this respect, clearance of the substance and protein binding are specifically important. Care should be taken to avoid *in vitro* conditions, which have influences on those parameters to avoid non-intended differences in tissue partitioning and in tissue exposure over time between *in vitro* and *in vivo*. Predictions for IVIVE based on measured intracellular concentrations in target organs rather than nominal concentrations of the *in vitro* datasets are critical for the validity of the predictions. *In vitro* observed effects may predict potential *in vivo* effects with a better predictivity if the *in vitro* effect can be considered being a step in the AOP. For the purpose of PBK modeling, generic modeling platforms might be preferable depending on the scenario which has to be solved.

## Data Availability

The data analyzed in this study are subject to the following licenses/restrictions: The article summarizes the data that have already been introduced in previous publication of the same authors. Requests to access these datasets should be directed to engi.algharably@charite.de.

## Supplementary Material

Supplementary Figure S1 BMD modeling for the dose-response curve of the amiodarone effect on ChAT activity in human brain cells (model averaging). BMD, benchmark dose; ChAT, choline acetyl transferase.

Supplementary Figure S2 Simulated (line) and observed (point) plasma concentration–time profiles of IBU, following oral administration of a single 600 mg dose in an elderly male volunteer using two modeling platforms. **(A)** MATLAB. Conditions: fu of 0.01; hepatic clearance of 0.06 L/h/kg bw (Greenblatt et al., 2008) and tissue/plasma partition coefficient of 3.01; **(B)** PK-Sim®. Conditions: fu of 0.01; hepatic clearance of 0.9 mL/min/kg (Greenblatt et al., 2008); specific intestinal permeability (Peff) of 6.3e-06 cm/s (optimized in silico); tissue/plasma partition coefficient model according to Schmitt (2008), compared to experimental data reported by Greenblatt et al. (2008).
