# Supplementary figures and images for: In Vitro–In Vivo Extrapolation by Physiologically Based Kinetic Modeling: Experience With Three Case Studies and Lessons Learned
Source: Front Toxicol. 2022 Jul 18;4:885843. doi: 10.3389/ftox.2022.885843 (PMC9340473; doi:10.3389/ftox.2022.885843)

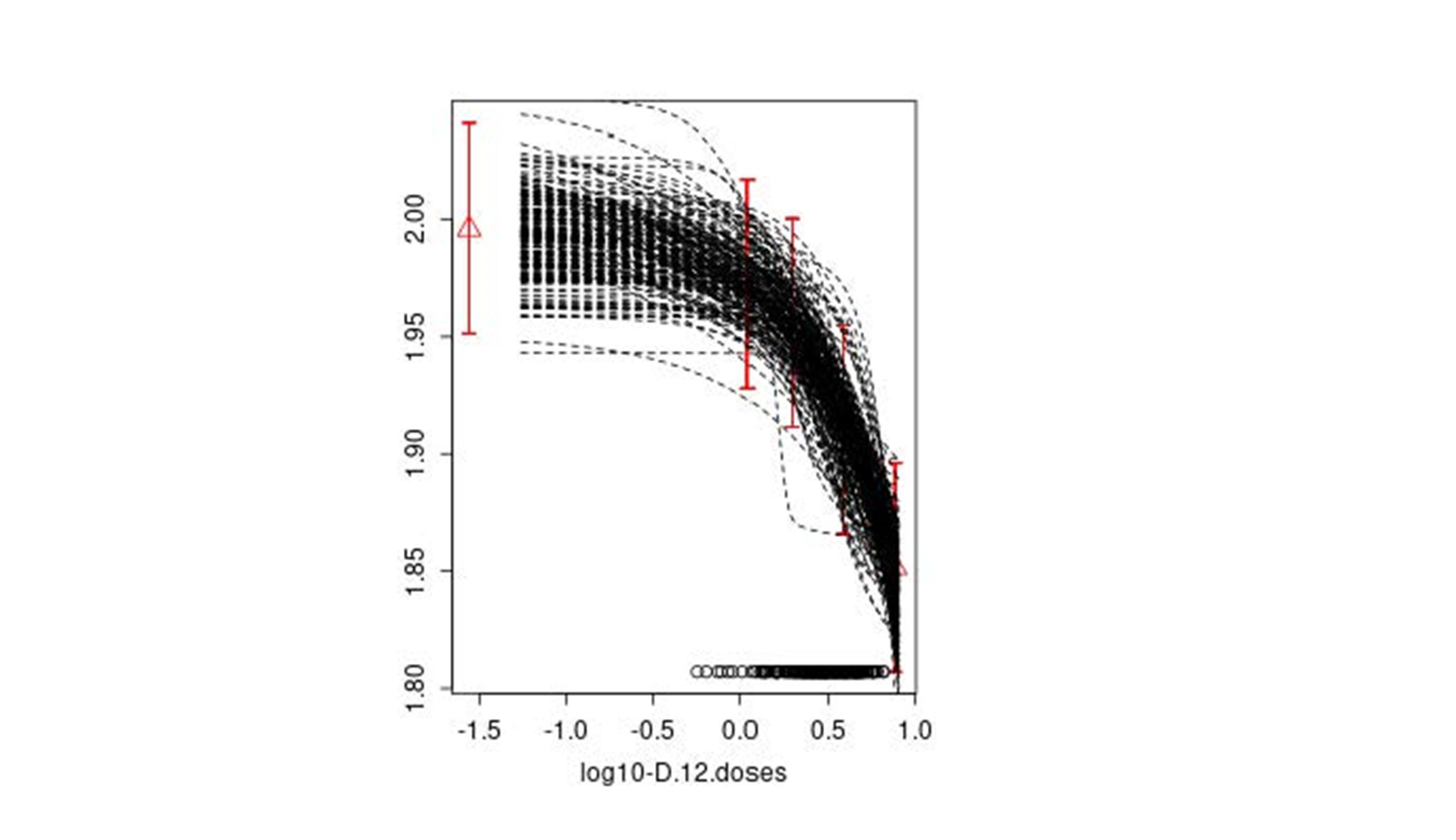

Supplement: Supplementary file 1 [file Image1.JPEG]

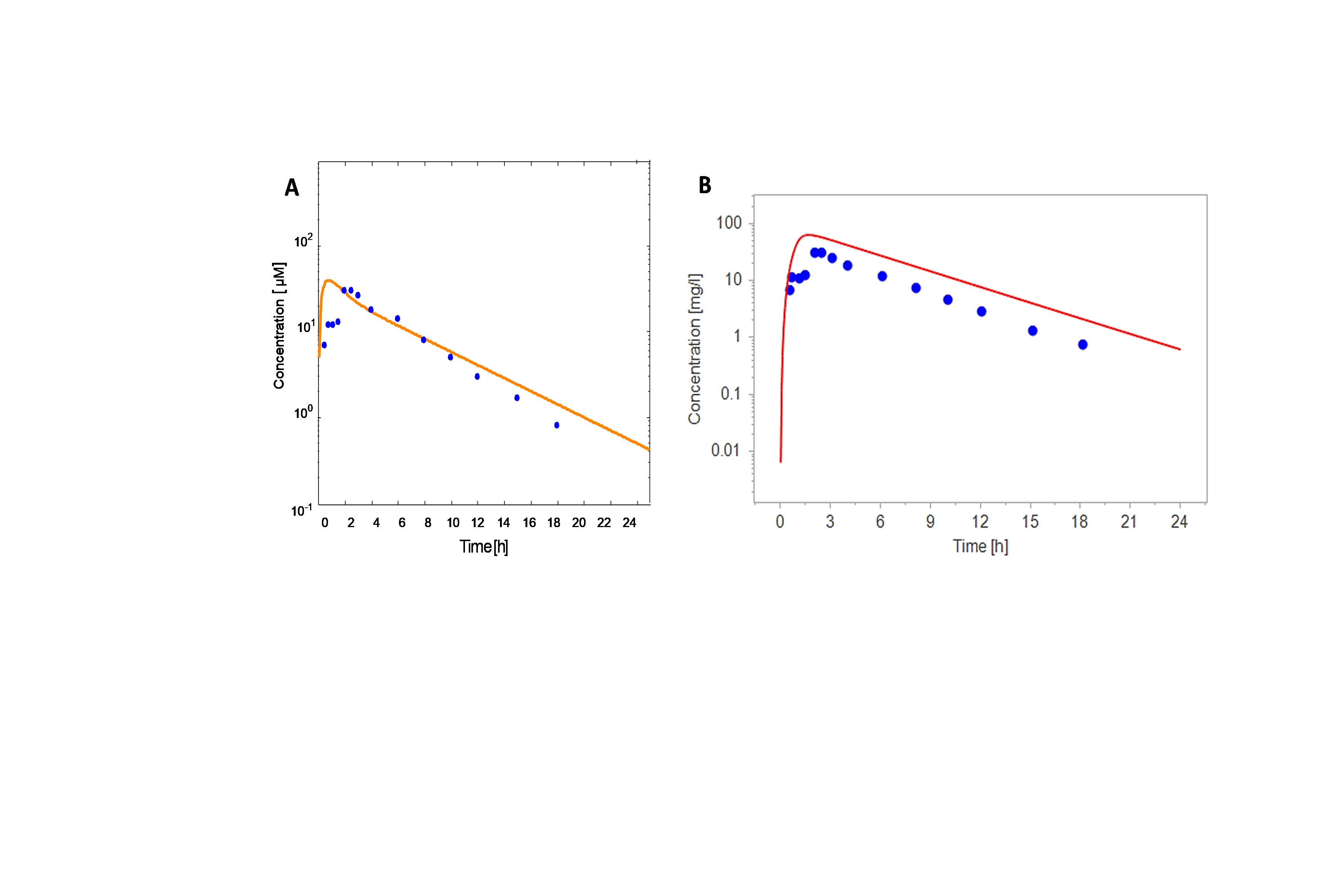

Supplement: Supplementary file 2 [file Image2.TIF]
